# Supplementary figures and images for: Genetic perturbation of mitochondrial function reveals functional role for specific mitonuclear genes, metabolites, and pathways that regulate lifespan
Source: GeroScience. 2023 Apr 22;45(4):2161–78. doi: 10.1007/s11357-023-00796-4 (PMC10651825; doi:10.1007/s11357-023-00796-4)

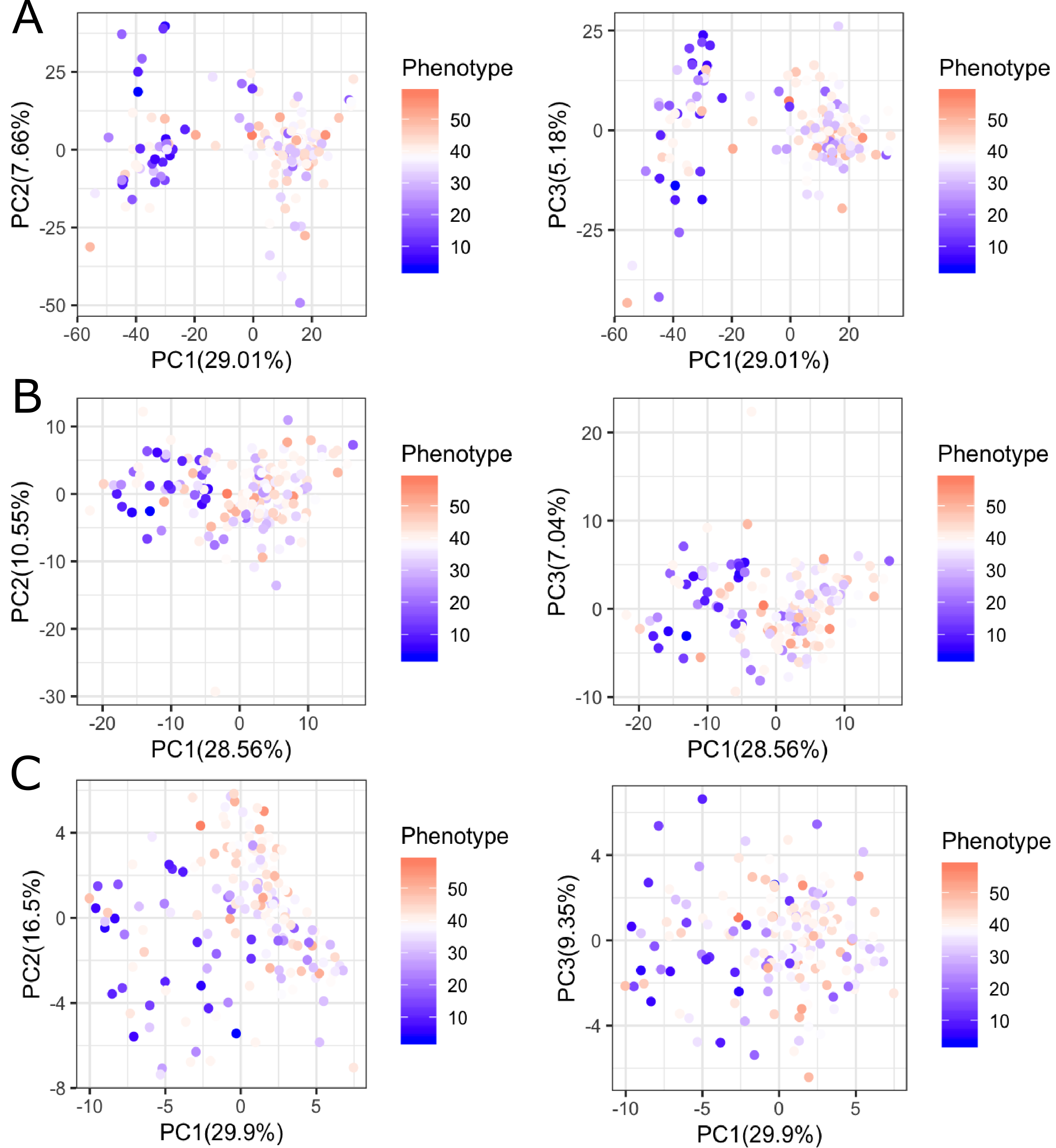

Supplement: Supplementary file 1 — Supplementary Figure 1 (PNG 420 KB) [file 11357_2023_796_MOESM1_ESM.png]

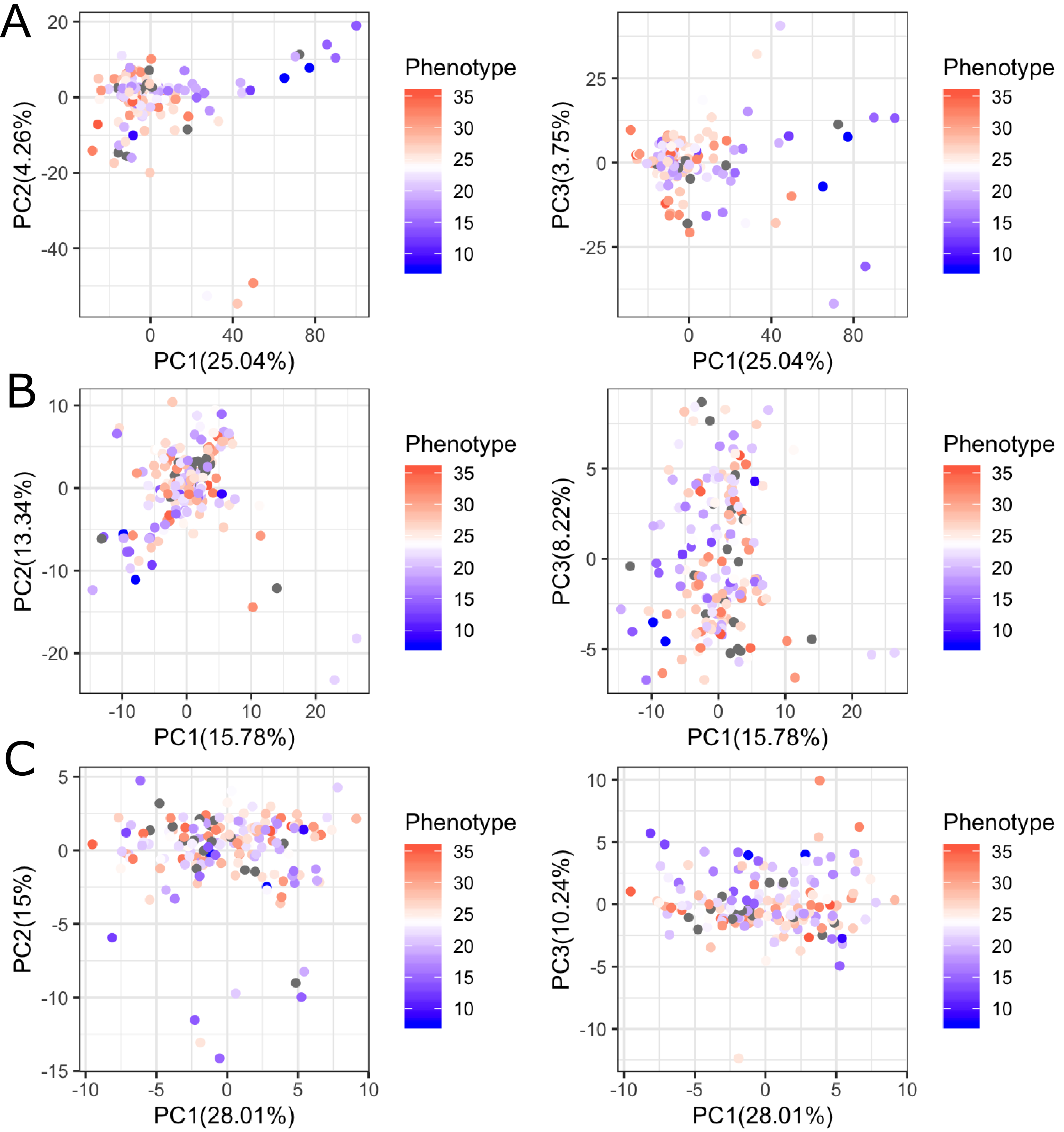

Supplement: Supplementary file 2 — Supplementary Figure 2 (PNG 391 KB) [file 11357_2023_796_MOESM2_ESM.png]
